# LGR4 and LGR5 Regulate Hair Cell Differentiation in the Sensory Epithelium of the Developing Mouse Cochlea

**DOI:** 10.3389/fncel.2016.00186

**Published:** 2016-08-05

**Authors:** Magdalena Żak, Thijs van Oort, Ferry G. Hendriksen, Marie-Isabelle Garcia, Gilbert Vassart, Wilko Grolman

**Affiliations:** ^1^Department of Otorhinolaryngology and Head and Neck Surgery, Brain Center Rudolf Magnus, University Medical Center UtrechtUtrecht, Netherlands; ^2^Institut de Recherche Interdisciplinaire en Biologie Humaine et Moléculaire, Faculty of Medicine, Université Libre de BruxellesBrussels, Belgium

**Keywords:** cochlea, development, hair cells, Wnt signaling, LGR4, LGR5

## Abstract

In the developing cochlea, Wnt/β-catenin signaling positively regulates the proliferation of precursors and promotes the formation of hair cells by up-regulating *Atoh1* expression. Not much, however, is known about the regulation of Wnt/β-catenin activity in the cochlea. In multiple tissues, the activity of Wnt/β-catenin signaling is modulated by an interaction between LGR receptors and their ligands from the R-spondin family. The deficiency in *Lgr4* and *Lgr5* genes leads to developmental malformations and lethality. Using the *Lgr5* knock-in mouse line we show that loss of LGR5 function increases Wnt/β-catenin activity in the embryonic cochlea, resulting in a mild overproduction of inner and outer hair cells (OHC). Supernumerary hair cells are likely formed due to an up-regulation of the “pro-hair cell” transcription factors *Atoh1, Nhlh1*, and *Pou4f3*. Using a hypomorphic *Lgr4* mouse model we showed a mild overproduction of OHCs in the heterozygous and homozygous *Lgr4* mice. The loss of LGR4 function prolonged the proliferation in the mid-basal turn of E13 cochleae, causing an increase in the number of SOX2-positive precursor cells within the pro-sensory domain. The premature differentiation of hair cells progressed in a medial to lateral gradient in *Lgr4* deficient embryos. No significant up-regulation of *Atoh1* was observed following *Lgr4* deletion. Altogether, our findings suggest that LGR4 and LGR5 play an important role in the regulation of hair cell differentiation in the embryonic cochlea.

## Introduction

Hearing is the process by which mechanical sound stimuli are transduced into electrical signals within the auditory nerve and transmitted to the cortex. The primary transducers of sound are the mechanosensory hair cells, residing within the sensory epithelia of the hearing organ, the cochlea.

The sensory epithelium is comprised of sensory hair cells and their surrounding auxiliary supporting cells. Both cell types originate from a progenitor pool of ‘pro-sensory’ cells all of which express the transcription factor SOX2, the Notch ligand Jagged1, and the cell cycle inhibitor p27^KIP1^ (Adam et al., 1998; Chen and Segil, 1999; Morrison et al., 1999; Kiernan et al., 2005; Brooker et al., 2006). Following terminal mitosis, a subset of pro-sensory cells up-regulate ATOH1, a bHLH transcription factor, and differentiates into hair cells (Bermingham et al., 1999; Zheng and Gao, 2000; Woods et al., 2004; Cotanche and Kaiser, 2010). Hair cells then instruct their neighbor cells to down-regulate ATOH1 and form the supporting cells (Lanford et al., 1999; Zine et al., 2001; Jeon et al., 2011).

Hair cells are very vulnerable and can be damaged by various factors, such as noise, ototoxic medication, and aging (Bodmer, 2008). An adult mammalian cochlea lacks regenerative potential and therefore hair cell loss is irreversible and leads to hearing loss. Recent studies, however, showed that a neonatal mouse cochlea possesses some regenerative capacities (revised in Atkinson et al., 2015).

Previous work suggests that some supporting cells possess regenerative capacity, mediated in part by the Wnt/β-catenin signaling pathway (reviewed in Atkinson et al., 2015; Jansson et al., 2015; Zak et al., 2015). The Wnt/β-catenin signaling pathway is a highly conserved signaling cascade controlling proliferation and hair cell formation in the developing inner ear (reviewed in Jansson et al., 2015; Zak et al., 2015). Wnt/β-catenin signaling induces hair cell formation by up-regulating the expression of *Atoh1*, the main “pro-hair cell gene” (Shi et al., 2010, 2014). Stimulating Wnt/β-catenin signaling pathway in the supporting cells induces their proliferation and differentiation into hair cells *in vivo* and *in vitro* (Chai et al., 2012; Shi et al., 2012, 2013; Jan et al., 2013). The supporting cells that show this regenerative capacities express LGR5, a stem cell and progenitor cell marker present during embryonic development and in self-renewing tissues (Barker et al., 2007, 2010; Jaks et al., 2008; Garcia et al., 2009; Chai et al., 2012; Chen et al., 2014, 2015; Jacques et al., 2012; Shi et al., 2012, 2013; Plaks et al., 2013; Takeda et al., 2013; Yee et al., 2013; Bramhall et al., 2014; Kawasaki et al., 2014; Miller et al., 2014; Ng et al., 2014; Ren et al., 2014; Sukhdeo et al., 2014; Song et al., 2015).

LGR5, also known as GPR49, is a member of the leucine-rich repeat-containing G-protein coupled receptors (LGRs) family, which is known for binding to their ligands from the R-spondin family to potentiate the activity of Wnt/β-catenin signaling pathway *in vitro* (Glinka et al., 2011; Ruffner et al., 2012; Carmon et al., 2014). In the fetal intestines, the lack of *Lgr5* expression up-regulates Wnt/β-catenin activity leading to precocious Paneth cell differentiation without detectable effects on the differentiation of other cell lineages or proliferation (Garcia et al., 2009). In the cochlea, the spatiotemporal expression pattern of LGR5 expression has been investigated (Chai et al., 2011; Shi et al., 2012), but the effects of *Lgr5* deficiency have not yet fully been addressed.

In multiple tissues, LGR5 is expressed in cells that are also positive for LGR4, an another member of the LGR family (Snippert et al., 2010; de Lau et al., 2011; Mustata et al., 2011; Kinzel et al., 2014; Ren et al., 2014). LGR4, also known as GPR48, is involved in the regulation of Wnt/β-catenin activity by playing a permissive role in the Wnt/β-catenin signaling pathway (Mustata et al., 2011). The lack of *Lgr4* expression decreases Wnt/β-catenin activity leading to hypoplasia and developmental defects in many tissues (Mustata et al., 2011; Sone et al., 2013; Wang et al., 2013; Kinzel et al., 2014). The expression and role of LGR4 in the developing cochlea has not yet been investigated.

In the present study, we investigated how the loss of LGR4 and LGR5 function affects Wnt/β-catenin activity in the developing mouse cochlea and whether the lack of *Lgr4* and *Lgr5* expression influences the proliferation and hair cell differentiation in the embryonic cochlea.

## Materials and Methods

### Animals

*Lgr4-LacZ* mice containing the LacZ knock-in allele at the *Lgr4* locus were on a CD1 background (Leighton et al., 2001; Mendive et al., 2006; Mustata et al., 2011). We used the hypomorphic *Lgr4-LacZ* mutant mice because they display a milder phenotype than the null mutant mice, which show growth retardation associated with embryonic and neonatal lethality (Kato et al., 2006). Hypomorphic heterozygous *Lgr4-LacZ* mice are healthy and fertile, while hypomorphic homozygous *Lgr4-LacZ* mice survive 4 weeks after birth (Mendive et al., 2006). Inserting the LacZ reporter gene into the *Lgr4* locus allows for easy examination of the spatial pattern of gene expression in tissue.

*Lgr5-eGFP* mice (Barker et al., 2007) containing the *EGFP-Ires-CreERT2* cassette knocked-in at the transcriptional start site of *Lgr5* were purchased from the Jackson Laboratory (Stock 008875) (Bar Harbor, Maine, ME, USA). Heterozygous *Lgr5-eGFP* mice are healthy and fertile, while homozygous *Lgr5-eGFP* mice die perinatally. Inserting *EGFP-Ires-CreERT2* cassette into the first exon of the gene enables colored labeling of cells that normally express Lgr5. *Lgr5-eGFP* mouse lines was on a C57BL/6 background.

C57BL/6JOlaHsd mice were obtained from Harlan Laboratories, Horst, The Netherlands.

For time breeding, females were examined daily for a vaginal plug. The day the plug was found was recognized as embryonic day 0.5 (E0.5), while the date of birth was recognized as postnatal day 0 (P0).

All animals had free access to both food and water and were kept under standard laboratory conditions. This study was carried out in accordance with the recommendation of the Animal Care and Use Committee of Utrecht University and the Local Ethical Committee of Université Libre de Bruxelles. All experimental procedures were approved by the Animal Care and Use Committee of Utrecht University (DEC 2013.I.04.048, DEC 2013.I.05.052, and DEC 2013.I.10.076) and by the Local Ethical Committee of Université Libre de Bruxelles (Ethical Protocol No. 534N).

### Genotyping

Transgenic mice were genotyped using genomic DNA isolated from pieces of ears and tails. Genomic DNA was extracted using DNeasy Blood and Tissue kit from Qiagen according to the manufacturer’s protocol. We used the following primer sets: *Lgr4* UpA: 5′-CCA GTC ACC ACT CTT ACA CAA TGG CTA AC-3′, *Lgr4* DownB: 5′-ATT CCC GTA GGA GAT AGC GTC CTA G-3′, *Lgr4* DownC: 5′-GGT CTT TGA GCA CCA GAG GAC-3′, *Lgr5* forward 5′-CTG CTC TCT GCT CCC AGT CT-3′, *Lgr5* wild type reverse 5′-ATA CCC CAT CCC TTT TGA GC-3′, *Lgr5* mutant reverse 5′-GAA CTT CAG GGT CAG CTT GC-3′.

### Cryosectioning and Whole Mount Sample Preparation

Heads from E14.5-E17, half sculls from P0-P1, and cochleae from P7-P42 mice were isolated in RNAlater and fixed in 2% paraformaldehyde (Sigma–Aldrich) in phosphate-buffered saline (PBS, pH 7.4) at room temperature for 1.5 h. Cochleae dissected from P7 and older mice were decalcified in 0.5 mM EDTA (Sigma–Aldrich) in PBS at 4°C for 2–4 days.

For cryosectioning, the cochleae were dehydrated by successively incubating them in 5, 10, and 25% sucrose (Sigma–Aldrich) in PBS at 4°C for 2 h. Next, the tissues were embedded in OCT compound (Sakura Finetek Europe B.V., Alphen aan Den Rijn, The Netherlands), cut into serial frozen sections of 10 μm thickness using Leica CM3050 cryostat and mounted on SuperFrost^∗^/plus microscope slides.

For whole mount samples, the otic capsule, the lateral wall, Reissner’s membrane, tectorial membrane and modiolus were removed using fine forceps. Next, the organ of Corti was dissected and micro-dissected into individual turns.

### Immunohistochemistry

Immunohistochemistry was performed on cryosections and cochlear whole mounts. The tissues were immersed in blocking solution consisting of 1% bovine serum albumin (BSA) and 0.1% triton X-100 (both from Sigma–Aldrich) in PBS for 30 min at room temperature. Next, specimens were incubated with primary antibodies at 4°C overnight. On the next day, tissues were rinsed with PBS and incubated with secondary antibodies at room temperature for 1 h. Both primary and secondary antibodies were diluted in 0.5% BSA in PBS. Afterward, tissues were again rinsed with PBS and mounted in Vectashield Antifade Mounting Medium with or without DAPI (Vector laboratories).

The following primary antibodies were used: anti-myosin7a (1:700; rabbit, Proteus Bioscience, 25-6790), anti-myosin7a (1:200; mouse, Developmental Studies Hybridoma Bank, 138-1-c), anti-SOX2 (1:200; goat, Santa Cruz Biotechnology, s-17320), anti-SOX2 (1:50, mouse, Millipore, MAB4343), anti-SOX2 (1:250, BD Pharmingen, 561469) anti-acetylated tubulin (ACTBA) (1:50; mouse, Sigma–Aldrich, T7451), anti-prestin (1:700; goat, Santa Cruz Biotechnology, N-20, sc-22692), anti-LGR4 (1:200; rabbit, Sigma–Aldrich, HPA030267, test of the antibody specificity shown in Supplementary Figure S1), anti-jagged1 (JAG1) (1:50; goat, Santa Cruz Biotechnology, C-20, sc-6011), anti-cyclinD1 (CCND1) (1:100; rabbit, Thermo Fisher Scientific, SP4, MA5-14512), anti-β-tubulin class III (TUJ1, 1:200; mouse, Covance, MMS-435P), anti-p75 NGF receptor (1:400; rabbit, Sigma–Aldrich, N3908), anti-MYO6 (1:300, Abcam, ab11096). Secondary antibodies were purchased from Molecular Probes Life Technologies. The following secondary antibodies were used: Alexa Fluor 488 donkey anti-rabbit (A21206), Alexa Fluor 488 donkey anti-mouse (A21202), Alexa Flour 594 donkey anti-rabbit (A21207), Alexa Flour 594 donkey anti-mouse (A21203), Alexa Flour 594 donkey anti-goat (A11058), Alexa Flour 405 goat anti-rabbit (A31556), Alexa Flour 405 goat anti-mouse (A31553).

For F-actin staining, after completing incubation with the secondary antibodies, tissues were rinsed in PBS and incubated with rhodamine phalloidin (1 μg/ml; Sigma–Aldrich, P1951) at room temperature for 1 h. Afterward, tissues were again rinsed with PBS and mounted in Vectashield Antifade Mounting Medium with or without DAPI (Vector laboratories).

Stained tissues were analyzed using a Zeiss LSM700 Scanning Confocal Microscope. Pictures were processed using Zen software (Carl Zeiss) and Photoshop (Adobe Creative Suite 5).

### β-Galactosidase Staining

Staining was performed on cryosections. Tissues were washed with 2 mM MgCl_2_ in PBS twice for 5 min each at room temperature and incubated in X-gal (4%) mixed with X-gal mixer (1:40, v/v) at 37°C for 2–4 h. X-gal mixer comprises 5 mM K_3_Fe(CN)_6_, 5 mM K_4_Fe(CN)_3_H_2_O, 0.01% sodium deoxycholate, 0.2% Nonidet NP-40, 2 mM MgCl_2_ (all from Sigma–Aldrich) in PBS. Next, tissues were rinsed with PBS and mounted in Mowiol (Sigma–Aldrich). Cell nuclei were stained with Nuclear Fast Red (Vector laboratories) according to the manufacturer’s protocol. Using this protocol, we stained cochleae from 3 *Lgr4-LacZ* knock-out and heterozygous mice. Control experiments found no non-specific staining in cochleae harvested from 3 wild type littermates.

### EdU Labeling

EdU (Invitrogen) was prepared in water at a concentration of 1 mg/ml. Pregnant females were injected intraperitoneally with 5 μg EdU per gram of body weight on day 13.5 post coitum. Females were injected three times with 2 h intervals and sacrificed 24 h after first injection. Next, heads of the embryos were collected and processed for cryosections. Sections underwent heat-induced antigen retrieval in sodium citrate buffer (10 mM, pH = 6) containing 0.05% Tween 20. Edu staining was performed using the Click-iT EdU Alexa Fluor 594 Imaging kit (Molecular Probes, Invitrogen) according to the manufacturer’s protocol.

### Plastic-Embedded Surface Preparations

Cochleae were isolated from *Lgr4-LacZ* mice at the age of P21–P24. We used three animals per genotype. Cochleae were perfused with trialdehyde [3% glutaraldehyde, 2% formaldehyde, 2.5% DMSO (all from Merck), 1% acrolein (Fluka), and 0.08M sodium cacodylate buffer (Sigma–Aldrich), pH 7.4] and then immersed in the same trialdehyde fixative for 3 h at room temperature. Next, cochleae were rinsed in 0.1 M sodium cacodylate buffer and decalcified in 10% EDTA (Sigma–Aldrich) pH 7.4 for 4–5 days at room temperature. Afterward, tissues were post-fixated with 1% osmium tetroxide (Sigma–Aldrich) and 1% potassium hexacyanoruthenate (II) hydrate (Alfa Aesar) for 2 h at 4°C and dehydrated with ethanol gradient. After being washed with 1,2-propylene oxide (Merck), cochleae were embedded in Spurr’s low-viscosity resin as previously described (Spurr, 1969).

Plastic-embedded cochleae were cut into two halves along the mid-modiolar plane, the half-turns were dissected and mounted onto glass microscope slides. Specimens were analyzed using a Zeiss Axiophot microscope equipped with DIC optics.

### Scanning Electron Microscopy

Cochleae were isolated from *Lgr4-LacZ* mice at the age of P21-P24. We used three animals per genotype. Cochleae were fixed with 2% glutaraldehyde in 0.1 M sodium cacodylate buffer (Sigma–Aldrich) pH 7.4 at 4°C overnight. On the next day, individual half-turns were micro-dissected and post-fixed using OTO method. Briefly, after being incubated with 1% osmium tetroxide (Sigma–Aldrich) for 30 min at room temperature, tissues were rinsed in water and incubated with 1% thiocarbohydrazide (Merck) for 30 min at room temperature. Next, tissues were again washed in water and incubated with 1% osmium tetroxide. Afterward, tissues were dehydrated with ethanol gradient and hexamethyldisilazane (Sigma–Aldrich). Dried specimens were placed on aluminum stubs covered with adhesive carbon disks (Agar Scientific) and analyzed using a Phenom Pro Scanning Electron Microscope.

### Cell Counting

Sox2-positive cells were counted in cryosections from E14.5 cochleae isolated from *Lgr4-LacZ* and *Lgr5-eGFP* mice. We counted Sox2-positive cells in the apical and mid-basal turns from three different animals per genotype.

We counted hair cells using either cochlear whole mounts obtained from P0 *Lgr5-eGFP* mice and stained with F-actin, or plastic-embedded surface preparations obtained from P21-P24 *Lgr4-LacZ* mice.

For cell counts in *Lgr5-eGFP* mice, we captured multiple images that covered the entire surface area of the specimens and then using Photoshop we constructed a composite picture encompassing the whole area of each whole mounted sensory epithelium. Next, using ImageJ we measured the entire length of each specimen and counted hair cells across a 450 μm region at 25, 50, and 75% positions along the length of the cochlear duct. Cochleae from 3 animals per genotype were analyzed.

For cell counts in *Lgr4-LacZ* mice, microscopic pictures were taken with a video camera and projected on an LCD-monitor to enable counting. The width of the screen corresponded to 150 μm of the specimen and the height corresponded to 110 μm. For each cochlea, the total length of the cochlear duct was calculated by adding the length of all the analyzed monitor projections. Next, the total number of hair cells across a 450 μm region at 25, 50, and 75% positions along the length of the cochlear duct was calculated. Cochleae from 3 animals per genotype were analyzed.

### Quantitative PCR

Total RNA was isolated from cochleae obtained from 3 to 6 animals using RNeasy Mini Kit and QIAshredder columns (Qiagen) according to the manufacturer’s instructions. Next, genomic DNA was removed using DNase I (Roche) and cDNA was synthesized using iSript cDNA Synthesis kit (Bio-Rad). qPCR reactions were performed with Quantifast Syber Green (Qiagen) according to the Qiagen real-time cycler conditions and using the iCycler MyiQ machine (Bio-Rad). Each PCR reaction was repeated in triplicate and the results were analyzed using iQ5 software (Bio-Rad). The relative quantification of gene expression was analyzed using the ΔΔCt method with *Gapdh* as the endogenous reference (Pfaﬄ, 2001).

The following primers were used: *Gapdh* forward 5′-AAC GAC CCC TTC ATT GAC-3′, *Gapdh* reverse 5′-TCC ACG ACA TAC TCA GCA C-3′, *Lgr4* forward 5′-CGA CTT CGC ATT CAC CAA CCT TTC-3′, *Lgr4* reverse 5′-CAA GTC CAG GGT TTC CAG GTT ATC-3′, *Lgr5* forward 5′-CGT TCG TAG GCA ACC CTT CTC TTA-3′, *Lgr5* reverse 5′-CGA GGC ACC ATT CAA AGT CAG TGT-3′, *Lgr6* forward 5′-CGC TAT CTT TGA ATG GTG CCA CTG A-3′, *Lgr6* reverse 5′-TTA TGA GAC AGC TCC AGG ATT CGG-3′, *Axin2* forward 5′-TGA CTC TCC TTC CAG ATC CCA-3′, *Axin2* reverse 5′-TGC CCA CAC TAG GCT GAC A-3′, *Pou4f3* forward 5′-GAA CCC AAA TTC TCC AGC CTA CAC-3′, *Pou4f3* reverse 5′-GTC GGG CTT GAA CGG ATG ATT CTT-3′, *Nhlh1* forward 5′-TGC AGA CAG ATG ACC CTT GA-3′, *Nhlh1* reverse 5′-ACT GAG CTC TGG GAA GCA GTT A-3′, *Cyclin D1* forward 5′-GTT CAT TTC CAA CCC ACC CTC -3′, *Cyclin D1* reverse 5′-AGA AAG TGC GTT GTG CGG TAG-3′, *Atoh1* forward 5′-TTT CCC CAA CTG CTT GAG AC-3′, *Atoh1* reverse 5′-TGC ATT GGC AGT TGA GTT TC-3′, *Lgr5* exon1 forward 5′-ACG ATT CTC TGC GCG GTC-3′, *Lgr5* exon1 reverse 5′- GGG CTG ATG CAG AAC CGA-3′.

### Statistical Analysis

**FIGURE 1:**
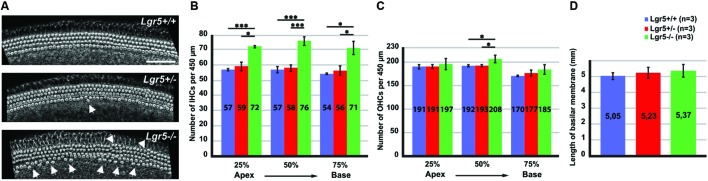
**Overproduction of hair cells in *Lgr5-eGFP* mice. (A)** A reconstruction of Z-stack images of the cochlear mid-basal turns stained with phalloidin. Homozygous *Lgr5-eGFP* mice showed overproduction of IHCs (arrows) and OHCs (arrowheads). **(B)** The number of IHCs was significantly increased along the cochlear duct in the homozygous *Lgr5-eGFP* mice. **(C)** The number of OHCs was also significantly increased in the mid-basal turn of the cochleae isolated from homozygous *Lgr5-eGFP* mice. **(D)** Comparing the length of basilar membrane between the *Lgr5* deficient mice and their wild type littermates.

**FIGURE 2:**
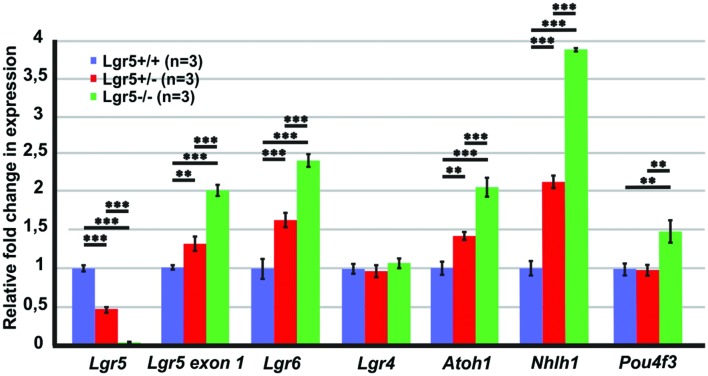
**qPCR results for the E13 cochleae of *Lgr5-eGFP* embryos.** The expression of *Lgr5* significantly decreased in the cochleae isolated from homozygous and heterozygous *Lgr5-eGFP* mice. The expression from *Lgr5* exon1 and the expression of *Lgr6*, *Atoh1, and Nhlh1* significantly increased in homozygous and heterozygous *Lgr5-eGFP* mice, while the expression of *Pou4f3* increased only in the homozygous *Lgr5-eGFP* mice. The expression of *Lgr4* was unaffected.

Statistics were conducted using Microsoft Excel (Microsoft) and SPSS 21 (IBM). The One-way ANOVA test or unpaired Student’s *t*-test was utilized with *p* < 0.05 considered statistically significant. The Tukey’s HSD or Games Howell *post hoc* test was applied. All data are presented as a group mean with standard deviation. Graphs were prepared using Adobe Illustrator (Adobe Creative Suite 5) and asterisks are used to show significance (^∗^ ≤ 0.05; ^∗∗^ ≤ 0.01; ^∗∗∗^ ≤ 0.001) in **Figures 1, 2, 6, 8**, and **10**.

## Results

### Homozygous *Lgr5-eGFP* Mice Generated Supernumerary Hair Cells and Supporting Cells

In the developing cochlea, LGR5 is expressed in the pro-sensory cells that are positive for Wnt/β-catenin activity and give rise to both hair cells and the supporting cells (Chai et al., 2011; Jacques et al., 2012).

To determine if loss of *Lgr5* affects hair cell differentiation, cochleae were isolated from newborn *Lgr5-eGFP* mice representing all three genotypes and stained with phalloidin to analyze the hair cell organization. In both heterozygous *Lgr5-eGFP* mice and their wild type littermates one row of inner hair cells (IHCs) and three rows of outer hair cells (OHCs) were evidenced (**Figure 1A**). Occasionally, misaligned IHCs were observed in heterozygous *Lgr5-eGFP* mice (**Figure 1A**). In contrast, homozygous *Lgr5-eGFP* mice showed pronounced misaligned hair cells and an overproduction of IHCs (**Figure 1A**).

This phenotype was analyzed along the whole cochlea. Hair cells were counted along the cochlear length at 25% of cochlear length (apical turn), 50% of cochlear length (mid-basal turn), and 75% of cochlear length (basal turn). The number of IHCs per 450 μm significantly increased at all three analyzed positions in homozygous *Lgr5-eGFP* mice as compared to their wild type and heterozygous *Lgr5-eGFP* littermates (**Figure 1B**). Additionally, the number of OHCs in the mid-basal region also increased from 192 ± 1.7 (*n* = 3) in wild type mice and 193 ± 1.5 (*n* = 3, *p* ≤ 0.05) in heterozygous *Lgr5-eGFP* mice to 208 ± 8.1 (*n* = 3, *p* ≤ 0.05) in homozygous *Lgr5-eGFP* mice, whereas the number of OHCs remained similar in the apical and basal turns in all genotypes (**Figure 1C**).

Then, we tested whether the supernumerary cells were formed due to abnormal cochlear convergence. If cochlear duct does not elongate properly, the sensory epithelium is shorter but the number of hair cells is unaffected. Since the hair cells do not have enough space along the cochlear duct, they form extra rows of hair cells (Montcouquiol et al., 2003; Wang et al., 2005; Qian et al., 2007). We compared the length of basilar membrane in *Lgr5* deficient mice and their control littermates, but no significant differences could be observed (**Figure 1D**). Thus, these data excluded that the supernumerary hair cells had resulted from a malfunction in cochlear convergence in homozygous *Lgr5-eGFP* (**Figure 1D**).

To determine whether differentiation of supporting cells was altered by *Lgr5* deficiency, we isolated the sensory epithelia from newborn *Lgr5-eGFP* littermates and stained them with p75 (NGFR), a protein exclusively expressed in the supportive inner pillar cells (IPCs) (Gestwa et al., 1999; Mueller et al., 2002; Doetzlhofer et al., 2009; Jahan et al., 2015). In the apical and mid-basal and basal turns of cochleae isolated from wild type mice and their heterozygous littermates, p75 was detected in the IPCs localized between three rows of OHCs and the single row of IHCs (Supplementary Figures S2A,A’). Similar staining pattern was observed in the cochleae harvested from homozygous *Lgr5-eGFP* mice (Supplementary Figure S2A”).

In the cochleae isolated from wild type mice and their heterozygous littermates, SOX2 was detected in a subset of supporting cells, including inner and outer pillar cells (IPCs and OPCs) and three rows of Deiters’ cells (Supplementary Figures S2B,B’). At the mid-basal turn of cochleae harvested from homozygous *Lgr5-eGFP* mice, four Deiters’ cells positive for SOX2 were localized beneath four OHCs (Supplementary Figure S2B”). These results imply that the supernumerary hair cells detected in the cochleae of homozygous *Lgr5-eGFP* mice were not formed at the expense of the supporting cells. Altogether, these data show that, at birth, *Lgr5* deficiency results in the over-production of both hair cells and supporting Deiters’ cells.

### *Lgr5* Deficient Mice Showed Increased Wnt/β-Catenin Activity Associated with Increased Expression of “Pro-hair Cell Genes”

In the fetal intestines, *Lgr5* deficiency up-regulates the Wnt/β-catenin signaling pathway causing precocious differentiation of Paneth cells (Garcia et al., 2009). To determine if the supernumerary hair cells observed in homozygous *Lgr5-eGFP* mice could be similarly related to an abnormal Wnt/β-catenin stimulation, we tested by qPCR the level of expression of several Wnt/β-catenin target genes in E13 cochleae (Chai et al., 2011).

First of all, the expression of *Lgr5* was tested using two different primer pairs. From the pair of primers spanning exons 8–10, we could confirm the genotypes with barely detectable expression of *Lgr5* in homozygous *Lgr5-eGFP* embryos (**Figure 2**). In contrast, with the use of a primer pair detecting exon 1 expression, which is upstream of the inserted cassette in the analyzed *Lgr5-eGFP* mouse model, we observed that transcription from the *Lgr5* promoter was increased by a fold change of 2.0 ± 0.06 (*n* = 3, *p* ≤ 0.001) in homozygous *Lgr5-eGFP* mice as compared to their wild type littermates (**Figure 2**).

The paralogue LGR6 receptor is also expressed in the pro-sensory domain and, *in vitro*, its expression is maintained by Wnt/β-catenin activity (Zhang et al., 2015). It is also transiently expressed in IPCs around birth (Zhang et al., 2015). We found that *Lgr6* expression was also increased by a fold change of 2.4 ± 0.1 (*n* = 3, *p*≤ 0.001) in homozygous *Lgr5-eGFP* mice whereas that of the third member of the Lgr family, the paralogue *Lgr4*, a non-Wnt/β-catenin target gene, was expressed at similar levels in the three genotypes (**Figure 2**).

We also tested expression of *Atoh1*, a reported Wnt/β-catenin target gene that induces formation of hair cells in the sensory and non-sensory cochlear epithelium (Bermingham et al., 1999; Zheng and Gao, 2000; Woods et al., 2004; Shi et al., 2010, 2014; Mulvaney et al., 2015). In homozygous *Lgr5-eGFP* embryos, *Atoh1* expression was significantly increased by a fold change of 2.1 ± 0.1 (*n* = 3, *p* ≤ 0.001) as compared to their wild type littermates (**Figure 2**). In addition, we tested the expression of two transcription factors *Nhlh1* and *Pou4f* that are necessary for formation and maintenance of hair cells (Xiang et al., 1997, 1998; Kruger et al., 2006). These factors are positively regulated by *Atoh1* in the developing mouse cerebellum, an *ex vivo* model of the developing mouse sensory epithelium, and in transfected mammalian cell lines (Klisch et al., 2011; Masuda et al., 2011; Ikeda et al., 2015). In the E13 homozygous *Lgr5-eGFP* cochleae, expression of *Nhlh1* and *Pou4f3* were significantly increased by a fold change of 3.9 ± 0.1 (*n* = 3, *p* ≤ 0.001) and 1.5 ± 0.1 (*n* = 3, *p* ≤ 0.01) as compared to their wild type littermates, respectively (**Figure 2**). Together, these results suggested that the lack of *Lgr5* expression leads to an up-regulation of Wnt/β-catenin activity in the pro-sensory cells of the embryonic E13 cochlea, resulting in an induction of “pro-hair cell genes” and consequently to supernumerary hair cell differentiation.

### The Loss of *Lgr5* Function Did Not Increase Proliferation

**FIGURE 3:**
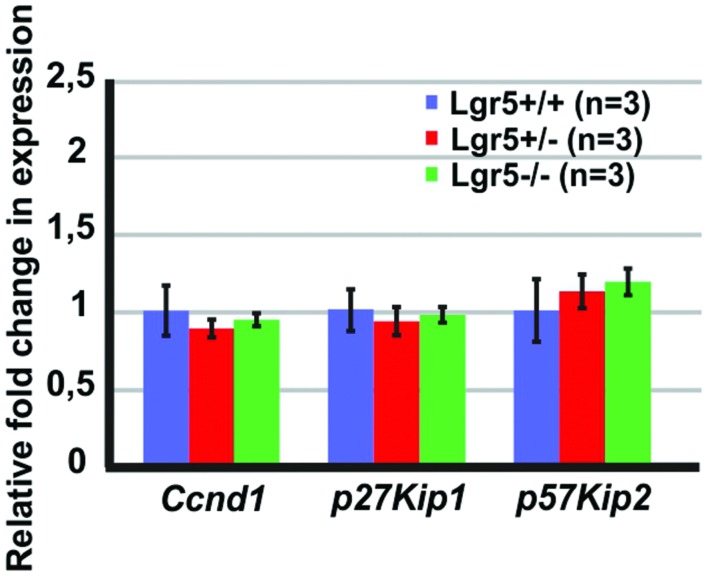
**The expression of cell cycle regulators in the E13 cochleae of *Lgr5-eGFP* embryos.** The expression of *Ccnd1*, *p27Kip1*, and *p57Kip2* did not change in the *Lgr5* deficient mice.

Next, we tested if the increased total number of cells could be associated with an increased proliferation. We tested the expression of *Cyclin D1* (*Ccnd1*), a gene required for cell cycle progression (Massague, 2004), but no significant changes could be detected (**Figure 3**). We also tested the expression of two cell cycle inhibitors, which are known to be expressed in the developing cochlea, *p27Kip1* and *p57Kip2* (Lowenheim et al., 1999; Lee et al., 2006; Liu and Zuo, 2008; Laine et al., 2010; Oesterle et al., 2011). The expression of *p27Kip1* and *p57Kip2* did not change in the cochleae of *Lgr5* deficient embryos (**Figure 3**). These results suggested that the supernumerary cells detected in homozygous *Lgr5-eGFP* mice are most likely not due to increased proliferation.

**FIGURE 4:**
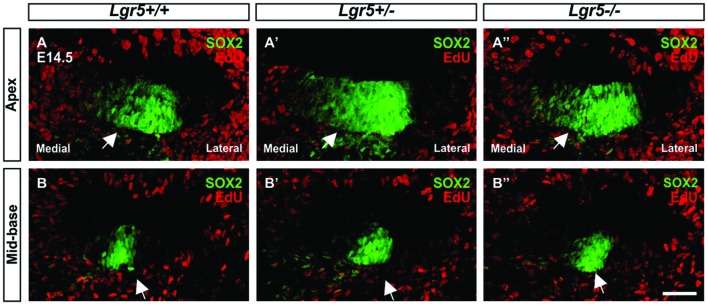
**Proliferation in the cochleae of E14.5 *Lgr5-eGFP* embryos. (A–B′′)** SOX2-positive pro-sensory cells (green, arrow) did not incorporate EdU (red). Cell nuclei were counterstained with DAPI (blue), scale bars indicate 25 μm.

To confirm this assumption, EdU labeling of proliferating cells was performed *in vivo* using *Lgr5-eGFP* mouse model. Pregnant females were injected three times with 2 h intervals at 13.5 days of gestation and sacrificed 24 h after first injection. In the E14.5 cochleae, EdU labeling was detected medial to pro-sensory domain in the apical turn and in mesenchyme surrounding the cochlear duct in wild type as well as heterozygous and homozygous *Lgr5-eGFP* mice (**Figures 4A–A**”). In the mid-basal turns, EdU-positive cells were observed only in mesenchyme outside the cochlear duct (**Figures 4B–B**”). None of the pro-sensory cells was EdU-positive, which confirms that the loss of *Lgr5* function did not increase proliferation.

### LGR4 Expression in the Developing Mouse Cochlea

In many other tissues, LGR4 is co-expressed with LGR5 in Wnt/β-catenin-responsive cells, in which the lack of *Lgr4* expression decreases Wnt/β-catenin activity (Snippert et al., 2010; de Lau et al., 2011; Mustata et al., 2011; Sone et al., 2013; Wang et al., 2013; Kinzel et al., 2014; Ren et al., 2014). We sought to investigate the spatiotemporal expression of LGR4 in the developing mouse cochlea to see if LGR4 is co-expressed with LGR5 in the same subset of cochlear cells. Our qPCR results obtained from E13 cochleae of *LGR5-eGFP* mice demonstrated that *Lgr4* is also expressed in the developing mouse cochlea. We used a collection of samples comprising six additional time points, representing different developmental events during cochlear development: E12 (proliferation), E17 (hair cell differentiation), P0 (birth), P8 (before onset of hearing), P21 (after onset of hearing), and P42 (adult cochlea). Our results confirmed that *Lgr4* and *Lgr5* are co-expressed in the developing mouse cochlea (Supplementary Figure S3A).

**FIGURE 5:**
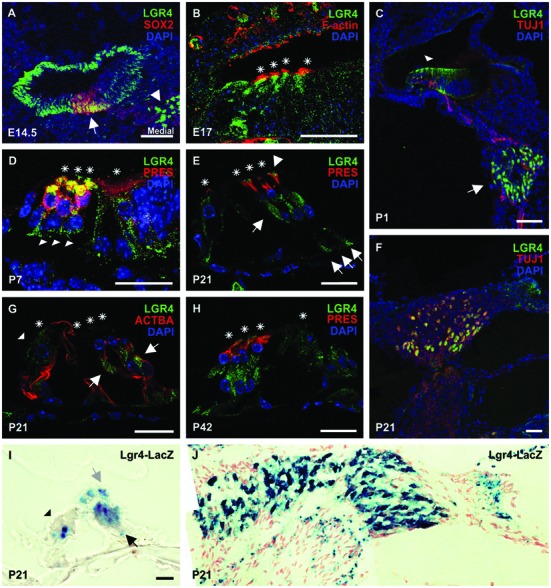
**The distribution of LGR4 in the developing mouse cochlea. (A)** At E14.5, strong LGR4 staining was detected in the cochlear duct and spiral ganglion cells (white arrowhead). LGR4 was detected in the pro-sensory domain, where it overlapped with SOX2 (white arrow), and non-sensory domains lateral and medial to the pro-sensory domain. **(B)** At E17, LGR4 (green) was detected in IHCs and OHCs (asterisks) and their surrounding supporting cells. Hair cells were labeled with phalloidin (F-actin) (red). **(C)** At P1, LGR4 expression was observed in the sensory epithelium (arrowhead) and spiral ganglion cells (arrow), from which projected TUJ1-positive nerves (red). **(D)** At P7, LGR4 was observed in hair cells (asterisks) that co-expressed Prestin (PRES) and in the surrounding supporting cells: Deiters’ cells (arrowheads) and IPCs and OPCs. **(E–G)** At P21, LGR4 was down-regulated in the OHCs and pillar cells, but was still expressed in the IHCs (arrowhead), Deiters’ cells (arrows), which were also positive for acetylated tubulin (ACTBA), and phalangeal processes of Deiters’ cells (arrowheads). LGR4 signals were also observed in the spiral ganglion cells. **(H)** At P42, LGR4 was detected in Deiters’ cells and their phalangeal processes. Cell nuclei were counterstained with DAPI (blue), asterisks indicate hair cells, scale bars indicate 20 μm in the picture **(B,D,E,G,H)** and 50 μm in **(A,C,F)**. **(I,J)** Cochlear cryosections from *Lgr4-LacZ* mice at the age of P21 were stained for β-galactosidase activity. Expression was seen in Deiters’ cells (black arrow) and their phalangeal processes (gray arrow), IHCs (black arrowhead), and spiral ganglion cells.

Firstly, we examined LGR4 expression in the developing cochlea using immunohistochemistry. At embryonic day E14.5, strong LGR4 signals were detected in the cochlear duct in the apical and mid-basal turns (**Figure 5A**; Supplementary Figure S1). LGR4 signals were detected not only in the pro-sensory domain, where it overlapped with SOX2, but also in the non-sensory domains flanking the pro-sensory domain and in mesenchymal cells medial to the cochlear duct (**Figure 5A**; Supplementary Figure S1). Additionally, LGR4 positive signals were observed in spiral ganglion cells throughout cochlear development, evidenced by comparing the expression of LGR4 with TUJ1, a marker for neural cells (**Figures 5A,C,F**; Supplementary Figure S1). In the cochlea at embryonic day E17, LGR4 was broadly distributed in the sensory epithelium with particularly strong staining in the differentiating hair cells, which were positive for F-actin (**Figure 5B**). At postnatal day P1, LGR4 was detected in the organ of Corti and in the greater epithelial ridge (**Figure 5C**). As the cochlea maturated, LGR4 expression decreased in the hair cells and became restricted to the Deiters’ cells. At P7, LGR4 was still strongly expressed in the hair cells, Deiters’ cells, as well as IPCs and OPCs (**Figure 5D**). At P21, LGR4 staining was detected in the IHCs and Deiters’ cells (**Figures 5E,G**). LGR4 expression was not detected in the OHCs because LGR4 staining did not overlap with prestin, a marker that specifically labels the OHCs. At P42, LGR4 was evident in Deiters’ cells that were localized beneath the OHCs labeled with prestin (**Figure 5H**).

To confirm the results obtained by immunohistochemistry, we performed X-gal staining on cochlear sections obtained from *Lgr4-LacZ* mice at the age of P21. In line with immunohistochemistry staining, the expression of the reporter gene was detected in the IHCs, Deiters’ cells, and spiral ganglion cells (**Figures 5I,J**).

To determine whether *Lgr4* and *Lgr5* are co-expressed in the developing mouse cochlea in the same cell subtypes, we applied immunohistochemistry on cochlear sections obtained from heterozygous *Lgr5-eGFP* mice. In the E17 cochlea of *Lgr5-eGFP* mice, LGR5-eGFP was detected in the entire organ of Corti, including hair cells and various supporting cells (Supplementary Figure S3B). At this time point, LGR4 was strongly expressed in the differentiating hair cells, where it co-localized with LGR5-eGFP and MYO7a (Supplementary Figure S3B). At P21, LGR5-eGFP strongly stained the IHCs and the third row of Deiters’ cells, but weaker LGR5-eGFP stains were also detected in the first two rows of Deiters’ cells (Supplementary Figure S3B). In the mature cochlea, LGR4 expression was also seen in the IHCs and all three Deiters’ cells, where LGR4 was co-expressed with LGR5-eGFP (Supplementary Figure S3B). These results indicated that LGR4 co-localized with LGR5 in the same type of cells in the developing cochlea.

### *Lgr4* Deficient Mice Generated Supernumerary OHCs and Deiters’ Cells

**FIGURE 6:**
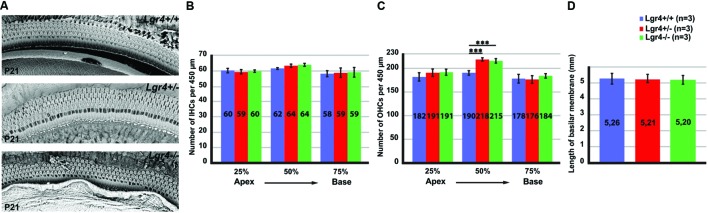
**Overproduction of hair cells in *Lgr4-LacZ* mice. (A)** Cochlear whole mounts captured by SEM. Both heterozygous and homozygous *Lgr4-LacZ* mice showed supernumerary OHCs. **(B,C)** Counting IHCs and OHCs along the cochlear duct showed a significant increase in the number of OHCs at 50% of cochleae obtained from heterozygous and homozygous *Lgr4-LacZ* mice. **(D)** Comparing the length of basilar membrane between the *Lgr4* deficient mice and their wild type littermates.

Next, we investigated if loss of LGR4 function could affect hair cell formation in the developing cochlea. Cochleae isolated from P21 *Lgr4-LacZ* littermates representing all three genotypes were analyzed by scanning electron microscopy (SEM). Wild type mice showed a clear pattern of three rows of OHCs and a single row of IHCs in the cochlea (**Figure 6A**). In contrast to their wild type littermates, heterozygous and homozygous *Lgr4-LacZ* mice showed supernumerary OHCs, usually organized in short patches forming a fourth row of OHCs (**Figure 6A**). The number of OHCs per 450 μm increased from 190 ± 0.5 (*n* = 3) in wild type mice to 218 ± 1.1 (*n* = 3) in heterozygous *Lgr4-LacZ* mice and 215 ± 1 (*n* = 3) in homozygous *Lgr4-LacZ* mice (*p* ≤ 0.001) (**Figure 6C**). Such phenotype was exclusively observed in the mid-basal region (**Figure 6C**). Moreover, in contrast to *Lgr5* deficient mice, the number of IHCs was similar in all *Lgr4-LacZ* genotypes (**Figure 6B**).

To see whether the supernumerary supporting cells were also produced in excess in the *Lgr4* deficient mice, whole mount preparations from P21 cochleae representing wild type and heterozygous *Lgr4-LacZ* mice were analyzed using Myosin7a (MYO7a) as a marker for hair cells, and phalloidin and acetylated tubulin (ACTBA) as markers for Deiters’ cells and pillar cells. In contrast to their wild type littermates (Supplementary Figure S4A), four OHCs positive for MYO7a were localized above four Deiters’ cells labeled with phalloidin and ACTBA in heterozygous *Lgr4-LacZ* mice, (Supplementary Figure S4B). This result implies that extra supporting cells were produced in addition to the supernumerary OHCs in the cochleae of *Lgr4* deficient mice.

Since the length of basilar membrane in the *Lgr4* deficient mice and their wild type littermates was not significantly different, we concluded that the fourth rows of OHCs and Deiters’ cells were not formed due to abnormal cochlear convergence (**Figure 6D**). These data would imply that the increased number of OHCs in the *Lgr4* deficient mice must have resulted from an increase in the total number of hair cells in the cochlea.

### *Lgr4* Deficient Mice Showed Premature Hair Cell Differentiation in a Medial-to-Lateral Gradient

**FIGURE 7:**
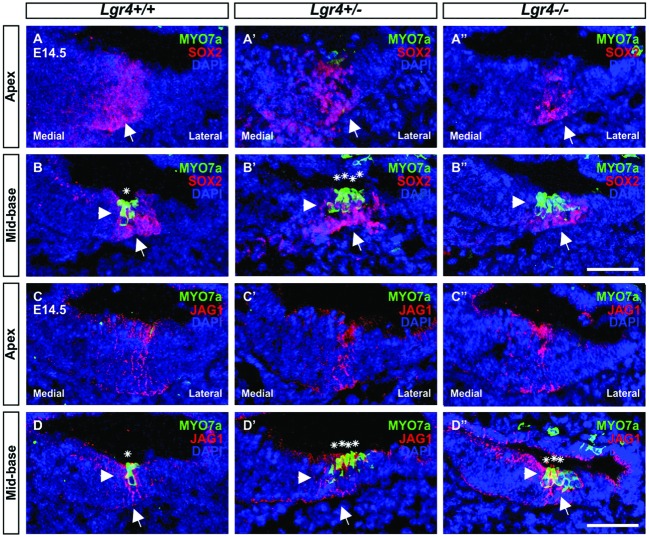
**Hair cell formation in the embryonic cochlea of *Lgr4-LacZ* mice. (A–D′′)** The cochlear cryosections obtained from *Lgr4* deficient mice and their wild type counterparts were stained for a hair cell marker (MYO7a, green) and two pro-sensory cell markers (SOX2, and JAG1, both red). Developing hair cells (asterisks) were detected only in the mid-basal turns. Cell nuclei were counterstained with DAPI (blue), scale bars indicate 20 μm.

To determine if extra OHCs in *Lgr4* deficient mice was associated with premature hair cell differentiation, cochleae from E14.5 *Lgr4-LacZ* embryo littermates were collected and co-stained for the pro-sensory markers *Sox2* or *Jag1* and *Myo7a*, one of the earliest hair cell markers (Self et al., 1998). In all the three genotypes, we detected MYO7a-positive hair cells in the mid-basal turn and no hair cells in the apical turn. (**Figures 7A–D”**), indicating that hair cells do not differentiate prematurely in a base-to-apex gradient in the *Lgr4* deficient embryos, However, since both heterozygous and homozygous *Lgr4-LacZ* embryos showed several rows of hair cells while their wild type controls had only a single one in the mid-basal turn (**Figures 7B,B’,B”,D,D’,D”**), these finding suggested that the hair cells could have prematurely differentiated in a medial-to-lateral gradient in the *Lgr4* deficient embryos.

### *Lgr4* Deficient Embryos Showed Increased Proliferation in the E13 Mouse Cochlea

**FIGURE 8:**
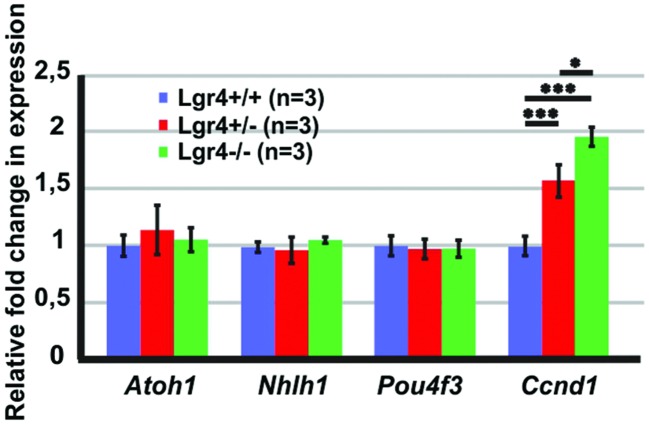
**The expression of “pro-hair cell genes” and cell cycle regulators in the E13 cochleae of *Lgr4* deficient mice.** The expression of *Atoh1*, *Nhlh1*, and *Pou4f3* did not change, while the expression of *Ccnd1* significantly increased in heterozygous and homozygous *Lgr4* mice.

To determine if the supernumerary cells observed in the *Lgr4* deficient mice was associated with increased expression of “pro-hair cell genes” in the pro-sensory cells, as it was detected in homozygous *Lgr5-eGFP* cochlea, qPCR experiments were performed on E13 cochlea. In contrast to *Lgr5* deficient embryos, the expression of *Atoh1* as well as that of the factors *Pou4f3* and *Nhlh1* was not increased in the L*gr4* deficient ones (**Figure 8**). Hence, the overproduction of hair cells did not result from the increased expression of genes required for the formation and maintenance of hair cells.

**FIGURE 9:**
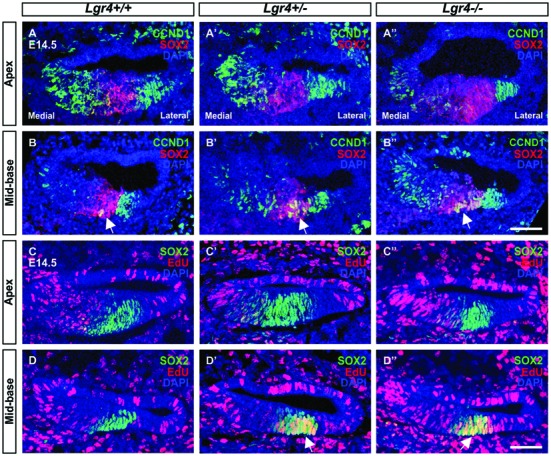
**Proliferation in the embryonic cochleae of *Lgr4* deficient mice. (A–B′′)** In the cochleae from heterozygous and homozygous *Lgr4-LacZ* mice, CCND1 (green) expression increased in the mid-basal turn, where CCND1 merged with red signal of SOX2 (arrows) in the pro-sensory domain. **(C–D′′)** The proliferation marker, EdU (red), was incorporated into pro-sensory cells (arrows) positive for SOX2 (green) in the mid-basal turn of the cochlea from *Lgr4* deficient mice. Cell nuclei were counterstained with DAPI (blue), scale bars indicate 20 μm.

Since supernumerary cell production may be associated with an increased proliferation in pro-sensory cells, we tested the expression of *Cyclin D1* (*Ccnd1*) in E13 cochleae. The expression of *Ccnd1* increased by twofold in *Lgr4* deficient embryos as compared to control littermates (**Figure 8**). These data were confirmed on cochlear sections obtained at the E14.5 stage by performing co-staining for SOX2-positive pro-sensory cells. In the apical turn, CCND1 was detected through the entire sensory epithelium irrespective of the genotype (**Figures 9A–B”**). In contrast, in the mid-basal turn, CCND1 was mainly seen lateral to the pro-sensory domain in wild type cochlea, whereas it was also additionally detected medial and within the pro-sensory domain in heterozygous and homozygous *Lgr4-LacZ* embryos (**Figures 9B’,B”**). Altogether, these results showed that the expression of CCND1 was increased in the mid-basal turn of the cochleae in *Lgr4* deficient embryos. Increased proliferation of pro-sensory cells in the cochleae of *Lgr4* deficient embryos was confirmed by EdU labeling of proliferating cells *in vivo*. *Lgr4-LacZ* females were injected three times with 2 h intervals at 13.5 days of gestation and sacrificed 24 h after first injection. In the E14.5 cochleae harvested from the wild type embryos, EdU-labeled cells were exclusively detected medial and lateral to the pro-sensory domain in the apical and mid-basal turns (**Figures 9C,D**). In heterozygous and homozygous *Lgr4-LacZ* embryos, EdU labeling was detected medial to the pro-sensory domain at the apical turn (**Figures 9C’,C”**). In the mid-basal turn of the cochlea from *Lgr4* deficient mice, EdU was incorporated into cells observed medial and lateral to the pro-sensory domain but also in the pro-sensory domain (**Figures 9D’,D”**). We counted SOX2-positive cells in the mid-basal turn of the cochleae isolated from *Lgr4-LacZ* embryos. The number of SOX2-positive cells increased from 22.7 ± 1.1 (*n* = 3) in wild type to 27.7 ± 2.1 (*n* = 3, *p* ≤ 0.05) in heterozygous *Lgr4-LacZ* and 27 ± 1.7 (*n* = 3, *p* ≤ 0.05) in homozygous *Lgr4-LacZ* embryos. Altogether, these data suggested that the loss of LGR4 function likely results in prolonged proliferative stage in Sox2-positive cells of the pro-sensory domain at the mid-basal turn, leading at later fetal stages to an increased number of OHCs and supporting cells.

### *Lgr4* Deficient Mice Showed Changes in the Expression of Some of Wnt/β-Catenin Target Genes

**FIGURE 10:**
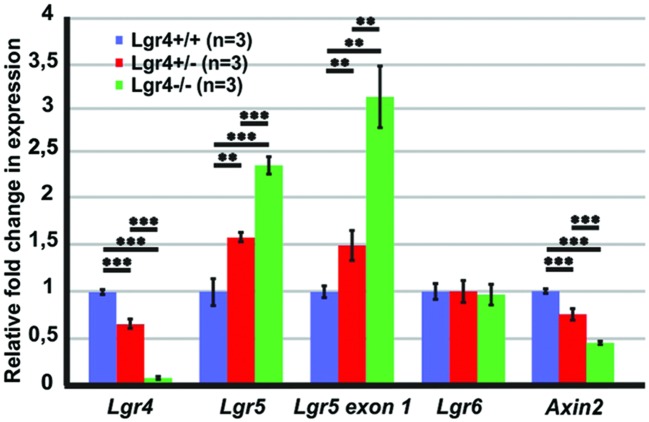
**The expression of Wnt/β-catenin target genes in the E13 cochleae of *Lgr4* deficient mice.** The expression of *Lgr4* significantly decreased in the cochleae of *Lgr4* deficient mice, while the expression of *Lgr5* and the expression from *Lgr5* exon1 significantly increased. The expression of *Lgr6* did not change, but the expression of and *Axin2* significantly decreased in heterozygous and homozygous *Lgr4* mice.

To see if the loss of LGR4 function affects Wnt/β-catenin signaling pathway in the developing cochlea, we tested the expression of Wnt/β-catenin target genes and LGR proteins in the cochleae of *Lgr4* deficient embryos at the age of E13 (**Figure 10**). Our qPCR results confirmed that the expression of *Lgr4* significantly decreased according to genotype and was barely detectable in the cochleae of homozygous *Lgr4-LacZ* embryos (**Figure 10**). In contrast, *Lgr5* expression (measured with the two different primer pairs), significantly increased by 2–3 fold change in the homozygous *Lgr4-LacZ* embryos as compared to their wild type littermates (**Figure 10**) whereas *Lgr6* expression was unaffected (**Figure 10**).

Since only one of the tested Wnt/β-catenin target genes showed changed expression in the cochleae of *Lgr4* deficient mice, we tested the expression of another Wnt/β-catenin target gene. *Axin2* is a Wnt/β-catenin target gene in the colorectal and liver tumor and in mammalian cell lines (Jho et al., 2002; Lustig et al., 2002). In the E14 mouse cochlea, *Axin2* is expressed in the mesenchymal cells surrounding the cochlear duct. On the medial site, mesenchymal cells are also positive for *Lgr4* (Supplementary Figure S1) and a fluorescent reporter of Wnt/β-catenin activity (Jacques et al., 2012). The expression of *Axin2* decreased by a fold change of 0.4 ± 0.1 (*n* = 3, *p* ≤ 0.001) in homozygous *Lgr4-LacZ* embryos as compared to their wild type littermates (**Figure 10**). Altogether, the results suggested that *Lgr4* deficiency changed the expression of some of the Wnt/β-catenin target genes in the cochleae of *Lgr4* deficient embryos.

## Discussion

In the developing cochlea, the Wnt/β-catenin signaling pathway regulates two important developmental events: the proliferation of precursor cells and their differentiation into hair cells (Jacques et al., 2012, 2014; Shi et al., 2014). During these two developmental events, the activity of Wnt/β-catenin signaling pathway is fine-tuned in a context dependent manner as the proliferation of precursors can be increased and prolonged by increasing Wnt/β-catenin activity during mitotic or post-mitotic phase, while hair cell differentiation can be increased only during a short time frame between E13.5 and E16 in mice (Jacques et al., 2012). Wnt/β-catenin signaling pathway stimulates hair cell differentiation in a base-to-apex gradient by up-regulating *Atoh1* expression, which is a gene necessary for hair cell formation and over-expressing *Atoh1* induces hair cell formation in the sensory and non-sensory regions of the cochlea (Bermingham et al., 1999; Zheng and Gao, 2000; Woods et al., 2004; Shi et al., 2010; Jacques et al., 2012; Shi et al., 2014; Mulvaney et al., 2015). Not much, however, is known about the molecular factors that regulate Wnt/β-catenin activity in the developing cochlea.

In multiple tissues, Wnt/β-catenin activity is regulated by LGR4 and LGR5 (Carmon et al., 2011; Glinka et al., 2011; Ruffner et al., 2012). LGR5 expression has already been investigated in the developing mouse cochlea (Chai et al., 2011; Shi et al., 2012), but the role of LGR4 and LGR5 in the regulation of Wnt/β-catenin activity, proliferation, and hair cell formation had not yet been addressed.

The current study showed that the lack of *Lgr5* function during development increases the number of hair cells and supporting cells in homozygous *Lgr5-eGFP* mice. This phenotype resembles effects of over-activating the Wnt/β-catenin signaling pathway during hair cell differentiation, a phenotype associated with increased *Atoh1* expression and resulting in the overproduction of hair cells and supporting cells (Jacques et al., 2012). In the cochleae of homozygous *Lgr5-eGFP* embryos, an increased expression of Wnt/β-catenin target genes *Lgr5*, *Lgr6*, and *Atoh1* was detected. The expression of *Pou4f3* and *Nhlh1*, which are “pro-hair cell genes” positively regulated by ATOH1, was also increased in the cochleae of homozygous *Lgr5-eGFP* embryos (Kruger et al., 2006; Jahan et al., 2010; Klisch et al., 2011; Masuda et al., 2011; Ikeda et al., 2015).

This is compatible with the reported increased expression of *Atoh1*, *Pou4f3*, and their downstream targets in the *Lgr5*-positive cells, which show high proliferation and hair cell regeneration capacities when isolated from the apical turn of the cochleae of P1-P2 *Lgr5-eGFP* mice (Waqas et al., 2016). Previous studies have shown that the increased expression of *Atoh1* results in the overproduction of IHCs along the entire length of cochlear duct in the hypomorphic *Sox2* mice, while the increased expression of all three “pro-hair cell genes,” *Atoh1*, *Pou4f3*, and *Nhlh1*, increases the hair cell number in the apical turn of the cochleae of mice with conditionally deleted *Neurod1* (Dabdoub et al., 2008; Jahan et al., 2010). Altogether, our data suggest that the loss of *Lgr5* expression in *Lgr5-eGFP* embryos up-regulates Wnt/β-catenin activity, which then increases expression of “pro-hair cell genes” leading to the overproduction of hair cells.

A similar phenotype has been observed in the intestines of *Lgr5-LacZ* homozygous embryos, in which the loss of LGR5 function increases Wnt/β-catenin activity leading to premature Paneth cell differentiation without increased cell proliferation (Garcia et al., 2009). This result suggests that LGR5 acts as a negative regulator of the Wnt/β-catenin signaling pathway in the fetal intestines (Garcia et al., 2009). The data presented in herein suggest that a similar mechanism exists in the developing mouse cochlea. Furthermore, studies using the LGR5-gain-of-function approach will further elucidate the role of LGR5 in regulating Wnt/β-catenin activity and hair cell differentiation in the developing cochlea.

Recently the expression of LGR6, a close homologue of LGR5, was reported in the subset of LGR5-positive cells (Chai et al., 2011; Shi et al., 2012; Zhang et al., 2015). Here, we detected LGR4, a third member of LGR family, in a wide range of cells including the LGR5-positive cells, which suggests that all three LGR proteins are expressed in the same subset of cochlear cells.

Loss of LGR4 function also increased the number of hair cells and supporting cells in the developing cochlea, but the *Lgr4* phenotype does not fully phenocopy the one from *Lgr5* deficient mice. The *Lgr4* deficiency increased the number of OHCs at the mid-basal turn, while the *Lgr5* deficiency resulted in the overproduction of IHCs along the cochlear length and OHCs at the mid-basal turn. In the E14.5 cochleae of *Lgr4* deficient mice, we observed premature hair cell differentiation in a medial-to-lateral gradient in the mid-basal turn, but the expression of “pro-hair cell genes” did not increase in the *Lgr4* deficient mice, which suggests that the overproduction of hair cells was induced by a different mechanism. An increased expression of *Ccnd1* and the increased incorporation of EdU in the mid-basal turn of the cochleae of *Lgr4* deficient mice were detected. These results implied that *Lgr4* deficiency prolonged the proliferation of cells in the pro-sensory domain, which resulted in the increased number of SOX2-positive precursors that gave rise to supernumerary OHCs and supporting cells.

Stimulating Wnt/β-catenin activity increases proliferation during the mitotic phase of cochlear development and results in cell cycle re-entry in the post-mitotic sensory epithelium (Jacques et al., 2012; Shi et al., 2013). Similarly to *Lgr5-eGFP* embryos, Wnt/β-catenin activity could have increased in *Lgr4* deficient mice leading to prolonged proliferation in the pro-sensory domain. However, *Lgr5* was the only Wnt/β-catenin target gene that increased the expression in the *Lgr4* deficient embryos, which could result from compensation between the LGR family members rather than from increased Wnt/β-catenin activity (Ruffner et al., 2012).

In the intestines, LGR4 is permissive to stimulate Wnt/β-catenin activity (Mustata et al., 2011), but analyses of the expression of Wnt/β-catenin target genes in the developing cochlea did not allow to conclude whether LGR4 plays the same role in the cochlea. The decrease in Wnt/β-catenin activity could have been masked by mesenchymal cells that surround the cochlear duct. The mesenchymal cells could send signals that compensate for the decrease in Wnt/β-catenin activity. Such a mechanism of compensation has previously been suggested to occur in the postnatal intestines of *Lgr4-LacZ* mice *in vivo* (Mustata et al., 2011). Therefore, further studies performed *ex vivo*, in absence of mesenchyme, would help to clarify if LGR4 is involved in the regulation of Wnt/β-catenin activity in the pro-sensory precursors.

Overall, we have demonstrated that LGR4 and LGR5 are important regulators of the cell differentiation in the sensory epithelium and the proliferation of precursors. We have also revealed their complex interaction with the Wnt/β-catenin signaling pathway in the developing cochlea. Further studies are necessary to fully understand the interaction between LGRs and their reported ligands from the R-spondin family. The loss of R-spondin 2 (Rspo2) function results in a phenotype similar to *Lgr4* deficient mice (Mulvaney et al., 2013), which suggests that RSPO2 could be binding to LGR4 and not to LGR5 and that LGR4 and LGR5 may play complementary functions in cochlear development.

## Author Contributions

MŻ was responsible for conceiving and designing the study, MŻ and TvO collected, assembled and analyzed the data, FH prepared cochlear cryosections and whole mounts, and carried out SEM analysis, M-IG and GV provided *Lgr4-LacZ* mice, MŻ, M-IG, and GV interpreted data and prepared the manuscript, WG provided administrative and financial support and approved the manuscript.

## Conflict of Interest Statement

The authors declare that the research was conducted in the absence of any commercial or financial relationships that could be construed as a potential conflict of interest.
