# Supplementary material for: LGR4 and LGR5 Regulate Hair Cell Differentiation in the Sensory Epithelium of the Developing Mouse Cochlea
Source: Front Cell Neurosci. 2016 Aug 5;10:186. doi: 10.3389/fncel.2016.00186 (PMC4988241; doi:10.3389/fncel.2016.00186)
Supplement: Supplementary file 1 [file Data_Sheet_1.DOC]

Supplementary Material

**LGR4 and LGR5 regulate hair cell differentiation in the sensory epithelium of the developing mouse cochlea.**

**Magdalena Żak^*^, Thijs van Oort, Ferry G. Hendriksen, Marie-Isabelle Garcia, Gilbert Vassart, Wilko Grolman**

**Corresponding author:**

M. Żak, ent-research@umcutrecht.nl, magdalena.zak1@gmail.com

## Supplementary Figures


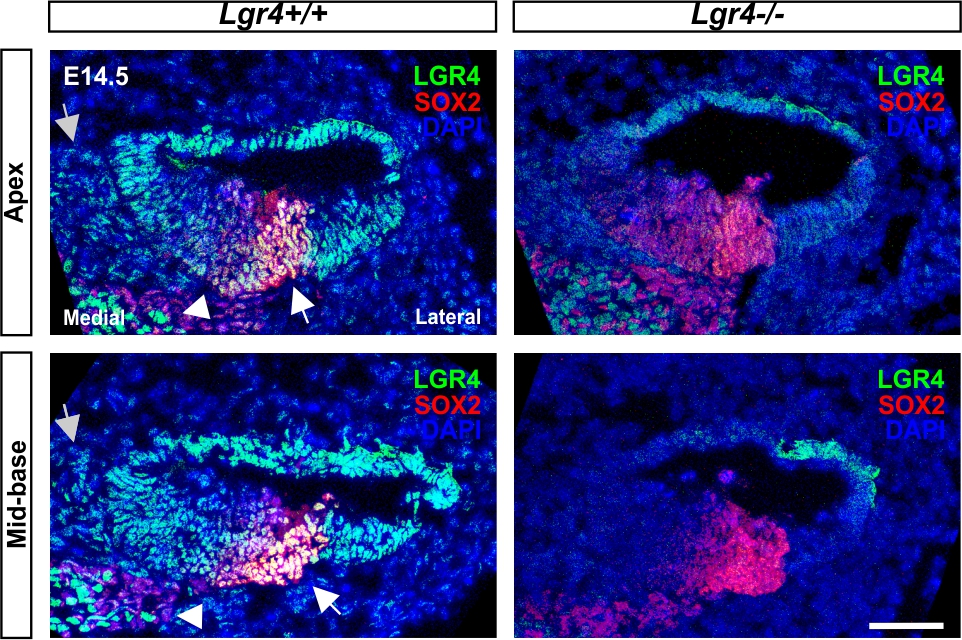


**Supplementary Figure 1**. **A test of the specificity of the anti-LGR4 antibody.** The cochlear cryosections obtained from E14.5 homozygous *Lgr4-lacZ* mice and their wild type counterparts were stained with the anti-LGR4 antibody and anti-SOX2 antibody, which is a marker for the pro-sensory domain. In the wild type embryos, anti-LGR4 antibody strongly stained cochlear duct in the apical and mid-basal turns. Anti-LGR4 antibody stained cells in the pro-sensory domain, where it overlapped with anti-SOX2 staining (white arrow). Additionally, anti-LGR4 antibody labeled cells in the non-sensory domains flanking the pro-sensory domain, spiral ganglion cells (white arrowheads), and mesenchymal cells medial to the cochlear duct (gray arrow). In the hypomorphic homozygous *Lgr4-lacZ* mice, anti-LGR4 staining visibly decreased compared to the wild type controls.


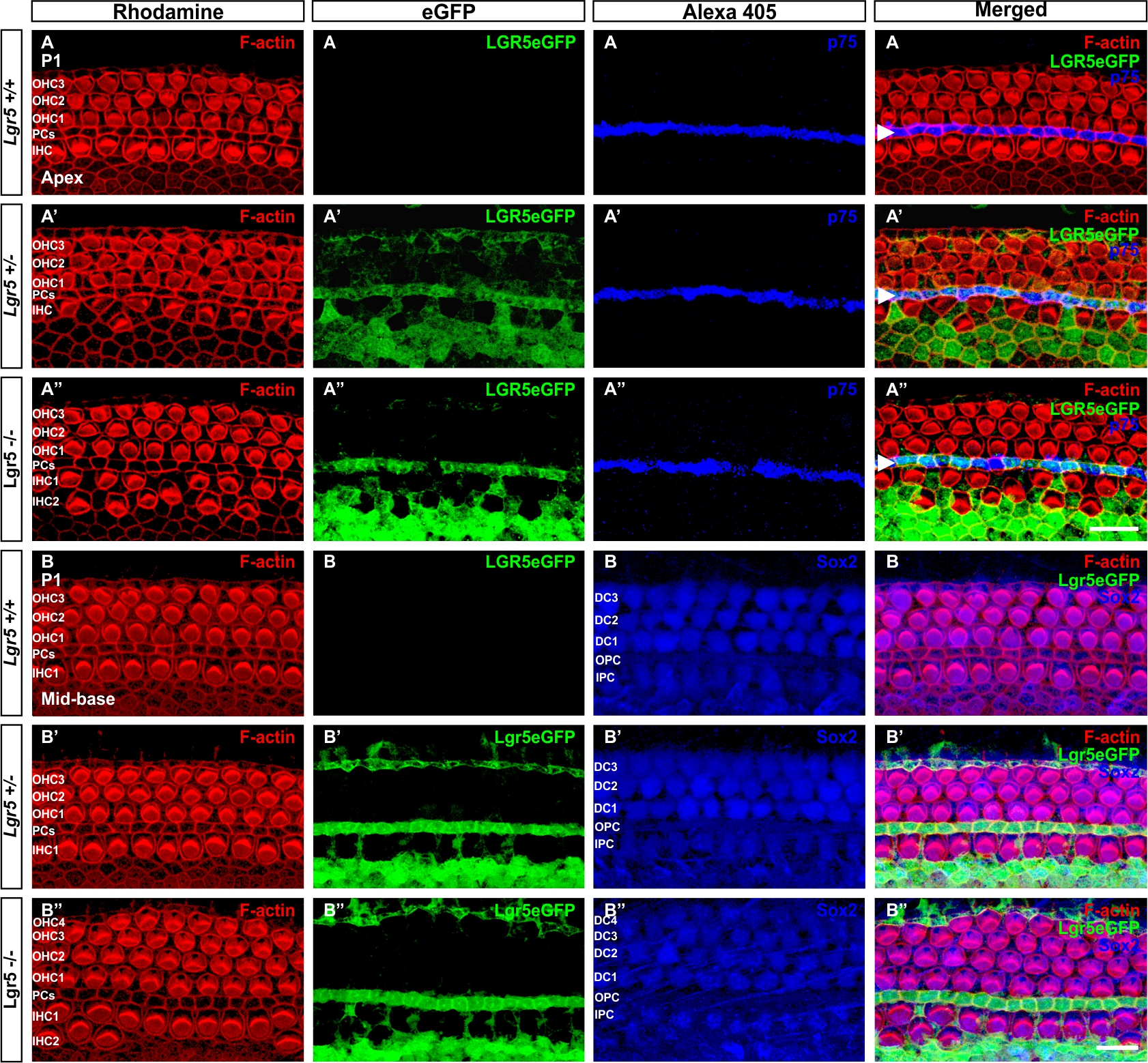


**Supplementary Figure 2**. **The supporting cells in** ***Lgr5-eGFP* mice.** A-A") At the apical turn in all three analyzed genotypes, inner hair cells and outer hair cells were positive for F-actin (red) and separated by pillar cells, which expressed LGR5-eGFP (green) and p75 (blue). In merged pictures, white arrows point at a clear line of pillar cells. B-B") Outer hair cells positive for F-actin (red) and Deiters' cells positive for SOX2 (blue) were produced in excess at the mid-basal turn in the cochleae of homozygous *Lgr5-eGFP* mice. Scale bars indicate 10 µm


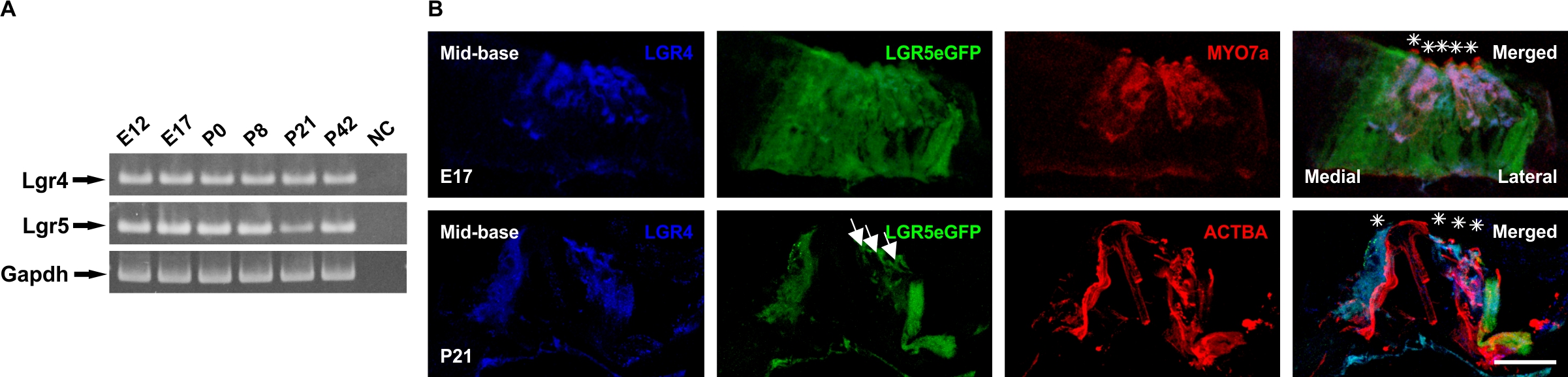


**Supplementary Figure 3**. ***Lgr4* was co-expressed with *Lgr5* in the developing mouse cochlea.** A) RT-PCR showed that *Lgr4* was co-expressed with *Lgr5* during cochlear development. B) At E17, LGR4 (blue) was co-expressed with LGR5-eGFP (green) in inner and outer hair cells (asterisks), which were labeled with MYO7a (red). At P21, LGR4 was co-expressed with LGR5-eGFP in inner hair cells and all three Deiters' cells (arrowheads), which were also positive for ACTBA (red). Cell nuclei were counterstained with DAPI (blue), scale bar indicates 20 µm.


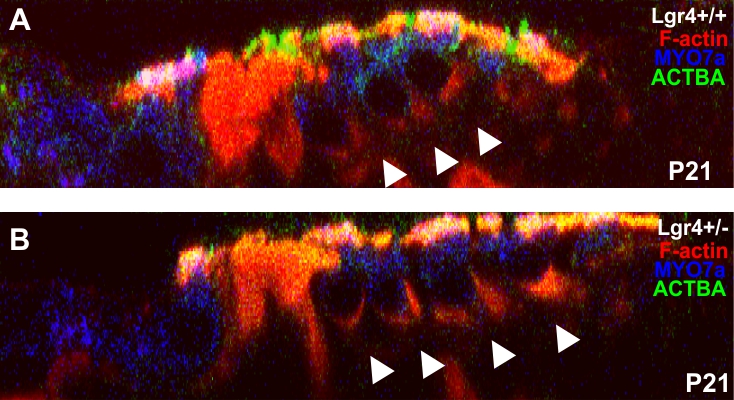


**Supplementary Figure 4**. **Supernumerary cells in *Lgr4-LacZ* mice.** A reconstruction of the Z-stack images of cochlear whole mounts obtained from a will type mouse (A) and its heterozygous *Lgr4-LacZ* littermate (B) showed that heterozygous mice had four outer hair cells positive for MYO7a (blue) and four Deiters' cells labeled with phalloidin (red) and ACTBA (green) (arrowheads).
